# Obtention of viable cell suspensions from breast cancer tumor biopsies for 3D chromatin conformation and single-cell transcriptome analysis

**DOI:** 10.3389/fmolb.2024.1420308

**Published:** 2024-08-22

**Authors:** Aura Stephenson-Gussinye, Luis A. Rendón-Bautista, Blanca E. Ruiz-Medina, Eduardo Blanco-Olais, Rosario Pérez-Molina, Cleofas Marcial-Medina, Yanin Chavarri-Guerra, Enrique Soto-Pérez-de-Celis, Andrea Morales-Alfaro, Ayerim Esquivel-López, Fernando Candanedo-González, Armando Gamboa-Domínguez, Rubén Cortes-González, Alejandro Alfaro-Goldaracena, Sara E. Vázquez-Manjarrez, Guido Grajales-Figueroa, Beatriz Astudillo-Romero, Jesús Ruiz-Manriquez, A. César Poot-Hernández, Paula Licona-Limón, Mayra Furlan-Magaril

**Affiliations:** ^1^ Molecular Genetics Department, Institute of Cellular Physiology, National Autonomous University of Mexico, Mexico City, Mexico; ^2^ Department of Cellular and Developmental Biology, Institute of Cellular Physiology, National Autonomous University of Mexico, Mexico City, Mexico; ^3^ Department of Hemato-Oncology, Instituto Nacional de Ciencias Médicas y Nutrición Salvador Zubirán, Mexico City, Mexico; ^4^ Department of Geriatrics, Instituto Nacional de Ciencias Médicas y Nutrición Salvador Zubirán, Mexico City, Mexico; ^5^ Department of Medicine, Division of Medical Oncology, University of Colorado Cancer Center, Denver, CO, United States; ^6^ Department of Pathology, Instituto Nacional de Ciencias Médicas y Nutrición Salvador Zubirán, Mexico City, Mexico; ^7^ Surgical Oncology Service, Department of Surgery, Instituto Nacional de Ciencias Médicas y Nutrición Salvador Zubirán, Mexico City, Mexico; ^8^ Department of Radiology, Instituto Nacional de Ciencias Médicas y Nutrición Salvador Zubirán, Mexico City, Mexico; ^9^ Department of Gastrointestinal Endoscopy, Instituto Nacional de Ciencias Médicas y Nutrición Salvador Zubirán, Mexico City, Mexico; ^10^ Unidad de Bioinformática y Manejo de Información, Institute of Cellular Physiology, National Autonomous University of Mexico, Mexico City, Mexico

**Keywords:** structural variations (SVs), Hi-C, single cell RNA sequencing, 3D genome architecture, breast cancer

## Abstract

Molecular and cellular characterization of tumors is essential due to the complex and heterogeneous nature of cancer. In recent decades, many bioinformatic tools and experimental techniques have been developed to achieve personalized characterization of tumors. However, sample handling continues to be a major challenge as limitations such as prior treatments before sample acquisition, the amount of tissue obtained, transportation, or the inability to process fresh samples pose a hurdle for experimental strategies that require viable cell suspensions. Here, we present an optimized protocol that allows the recovery of highly viable cell suspensions from breast cancer primary tumor biopsies. Using these cell suspensions we have successfully characterized genome architecture through Hi-C. Also, we have evaluated single-cell gene expression and the tumor cellular microenvironment through single-cell RNAseq. Both technologies are key in the detailed and personalized molecular characterization of tumor samples. The protocol described here is a cost-effective alternative to obtain viable cell suspensions from biopsies simply and efficiently.

## Introduction

In breast cancer, biopsy collection using thick needles is a standard diagnostic method. Core needle biopsies are used to characterize molecular markers related to the cancer subtype, inform treatment and provide prognostic information (Pagni et al., 2014; Ziv et al., 2016). However, the use of biopsies is not limited to the clinic as they also present a significant opportunity in research for “*de novo*” molecular characterization through genomic, and/or transcriptomic techniques. While surgical specimens often provide a larger amount of tissue, these may not always be available in breast cancer due to the increasing use of up-front systemic therapy before surgical excision, posing a challenge for obtaining untreated tumor tissue at the time of resection (Prat et al., 2015).

The molecular characterization of cancer is relevant due to the high complexity and heterogeneity of the disease and new technologies may allow for a better understanding of its various pathological processes. Recent techniques for cancer analysis include chromosome conformation capture technologies (Jia et al., 2017). These techniques enable the characterization of different levels of three-dimensional genome organization but have also proven to be valuable tools for detecting chromosomal structural variations (SVs) that may be difficult to characterize otherwise (Harewood et al., 2017; Stephenson-Gussinye and Furlan-Magaril, 2023).

Chromosomal SVs are key markers in various cancer types. In B-cell acute lymphoblastic leukemia, gene fusions resulting from chromosomal rearrangements such as translocations between chromosomes 9–22 (Philadelphia chromosome) or 12–21, are associated with incidence and prognosis in children (Woo et al., 2014; Mallard et al., 2022). SVs can also cause alterations in chromatin architecture, translating into enhancer-hijacking events and disruptions in gene expression, leading to tumoral progression (Wang et al., 2021; Wang et al., 2022b; Xu et al., 2022; Stephenson-Gussinye and Furlan-Magaril, 2023). These topological alterations have been observed in various malignancies involving oncogene expression and tumor-specific transcriptional programs (Liu T. et al., 2023; Xie et al., 2024).

Assessing the tridimensional genome organization may not be sufficient to characterize molecular signatures fully, and gene expression profiling is also indispensable to relate genomic structural variants to functional transcriptomic alterations and to identify new targets for cancer characterization, prognosis, or therapy.

Single-cell RNA sequencing (scRNA-seq) is a technology that allows transcriptomic characterization of tumors. This technique has several benefits over total RNA-seq as it identifies and characterizes the different cellular populations present in the tumor sample. Additionally, it does not require large cell numbers and provides a more precise approach to characterizing the tumor microenvironment (Zhang et al., 2021b). However, a huge bottleneck in its success when working with tumor samples is the requirement of clean (debris-free) single-cell suspensions with 70% or more viability, which is difficult to obtain from most biopsies. This is due to different factors such as sample heterogeneity, handling time, reduced cell recovery and low cell viability from tumoral cells that may have hypoxic or necrotic sections, among others (Roma-Rodrigues et al., 2019).

Applying both Hi-C and scRNA-seq technologies to the same patient sample allows for detailed molecular characterization of the tumoral cells and could potentially allow large-scale characterization of other pathologies. The processing of small amounts of tissue through tumor biopsy samples to obtain highly viable cell suspensions for multi-omic analysis protocols creates a wide range of opportunities for cancer research and could also be applied to other diseases. Here we present a detailed protocol that produces clean and viable cell suspensions to perform Hi-C and scRNA-seq from breast tumor biopsies.

## Materials and methods

Given the considerable tumor heterogeneity in some cancer types, the personalized molecular characterization of the cell population is crucial to understand cancer pathogenesis. Furthermore, the option to conduct multi-omic analyses derived from biopsies provides a new tool for cancer studies, particularly as obtaining naive tumor samples during surgeries is not always possible as patients might be already undergoing treatment that may alter the tumor environment. However, the molecular characterization of tumoral biopsies is challenging and requires tissues to be efficiently cryopreserved until enough samples are collected to process them with the scRNA-seq protocol, which uses a chip that accommodates a minimum of up to four samples. These factors pose significant obstacles, especially when protocols require viable and clean cell suspensions. For these reasons, we optimized an affordable protocol to obtain cell suspensions from primary breast cancer core needle biopsies, ensuring sufficient cell number and viability to conduct chromosomal conformation capture analysis by Díaz et al. (2018), and transcriptomic single-cell analysis with scRNA-seq (10X Genomics).

### Acquisition and storage of breast cancer biopsies

Primary breast cancer core needle biopsies and surgical samples were collected from female patients diagnosed with breast cancer, treated at the Instituto Nacional de Ciencias Médicas y Nutrición Salvador Zubirán in Mexico City (INCMNSZ) (n = 30). The study was approved by INCMNSZ’s research and ethics committee (Ref. 3274), and informed consent was obtained from all participants. For core biopsies, three cores per patient were taken using TruCut needles (BIOCORE MG 12G x 10CM, Histo) and placed in 5 mL of RPMI 1640 medium (Gibco) enriched with 10% fetal bovine serum (FBS, Gibco) and 1% ampicillin/streptomycin (Biowest). The sample was immediately placed on ice and transported to the laboratory in an insulated compartment. Surgery samples were collected from systemic treatment-naive patients undergoing surgery. After review by a pathologist, a tissue sample was provided and handled using the same methods. The size of the sample varied depending on tumor size and tissue availability after pathology analyses. The adjacent tissue was collected following the same method used for the tumors, ensuring it was taken from at least 1 cm away from the tumor-affected area and presented a macroscopically healthy appearance.

### Pre-freeze tissue dissociation protocol

**FIGURE 1:**
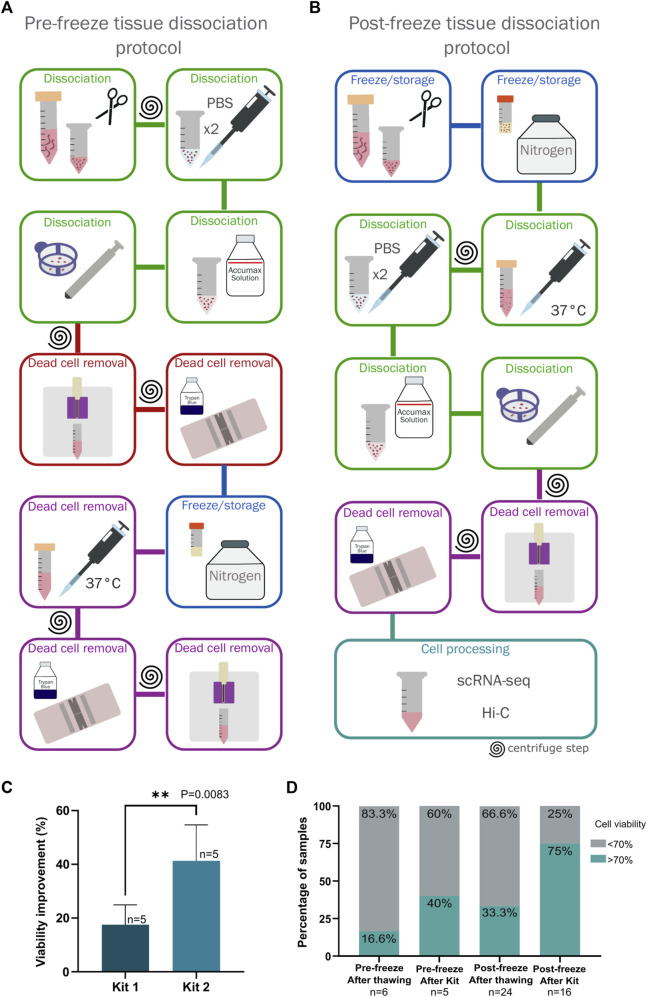
Comparison of two protocols for dissociation and freezing of breast cancer tumor biopsies. **(A)** Pre-freeze tissue dissociation protocol. The steps carried out to perform this protocol are indicated, highlighting in red the steps that were eliminated in the standardization process for the final protocol. **(B)** Post-freeze tissue dissociation protocol. The steps carried out in this protocol are indicated, this was established as the definitive protocol for processing and storing breast cancer tumor samples. **(C)** Comparison of cell viability using two dead cell removal kits. Kit 1 corresponds to the EasySep Dead Cell Removal Annexin V Kit (Stemcell technologies #17899) and Kit 2 corresponds to the Dead Cell Removal Kit (Miltenyi Biotec #130090101). Viability improvement was measured by subtracting the viability observed after one use of the kit minus viability observed before the kit processing. The difference between the kits was statistically significant via an unpaired *t*-test (*p* = 0.0083, n = 5). **(D)** Comparison of cell viability between the two tested protocols (Pre-freeze and post-freeze tissue dissociation protocols) comparing cell viability obtained immediately after thawing and disaggregating the sample (in the post-freeze protocol) and viability after using the dead cell removal kit.

A tissue processing protocol was initially standardized based on previously published recommendations (Lu et al., 2020b). Briefly, the sample was weighed, washed with PBS and dissociated to obtain a cellular suspension, which was enriched to a viability above 70% using a Dead Cell Removal Kit, and subsequently frozen and stored in liquid nitrogen. Later, the sample was thawed based on previously published recommendations (Lu et al., 2020b) for processing through Hi-C and scRNA-seq (Figure 1A). The cell viability was often low after thawing, consequently, a second use of the Dead Cell Removal Kit was necessary to increase cell viability. This resulted in a high cell loss.

### Post-freeze tissue dissociation protocol

#### Freezing and storage

Based on the previous observations, we implemented a new protocol that minimized sample manipulation and centrifugation steps (Figure 1B) to obtain the maximum number of cells while maintaining sufficient cell viability for subsequent experimental processing.

Upon receiving the sample, the tissue was weighed and then stored according to a previously published protocol with some minor modifications (Wu et al., 2021b). Briefly, the tissue was cut into 1 mm^3^ pieces, placed in 500 µL freezing medium containing 90% FBS (Gibco) with 10% dimethyl sulfoxide (DMSO, Sigma) in a 1.8 mL cryovial (Thermo Scientific), and frozen using a Mr. Frosty™ Freezing Container (ThermoFisher) at −80°C for 24–72 h before being transferred to liquid nitrogen (Figure 1B). The steps described below constitute a cost-effective alternative to the protocol described by Wu et al., which uses the Human Tumour Dissociation Kit (Miltenyi Biotec) following the manufacturer´s instructions (Wu et al., 2021b).

#### Sample dissociation

The tissue was thawed using a water bath at 37°C for a few seconds, transferred to a 15 mL tube and washed with 14 mL of pre-warmed 10% FBS-RPMI added dropwise. The sample was then centrifuged at 300 *g* for 10 min, the supernatant was discarded, and the tissue was placed in a 1.5 mL tube with PBS (Sigma). The sample was washed twice with 1 mL of cold PBS by gently tapping the tube and then allowing the tissue to settle for 2 min on ice to remove most blood and the remaining medium (Figure 1B).

To obtain a single cell suspension, PBS was removed and the Accumax solution (Sigma) was added using 100 µL per 0.01 g of tissue. The tissue was incubated at room temperature for 30 min, gently mixing at 10-min intervals. After the incubation period, the enzymatic digestion was stopped by adding 1 mL of RPMI medium with 10% FBS, and the cell suspension was passed through a 40 µm filter using a syringe plunger to apply mechanical disaggregation. We recovered a single-cell suspension free of large debris (Figure 1B).

#### Dead cell removal and measurement of cell viability

Cell viability was calculated using Trypan Blue (Sigma). Since subsequent protocols required a recommended viability of over 70%, the enrichment of live cells represented a crucial step in the protocol. Two dead cell removal kits were tested in 5 surgical samples for this step:

• Dead Cell Removal Kit (Miltenyi Biotec #130090101) using MS Columns with magnetic beads (Miltenyi Biotec #130042201) following the manufacturer’s instructions.
• EasySep Dead Cell Removal AnnV Kit (Stemcell technologies #17899) following the manufacturer’s instructions.

Both kits process cell suspensions through established protocols. However, a significant difference in the viability was observed between the two kits (Figure 1C). Measuring the viability of 5 tumoral samples before and after processing for each kit, the Dead Cell Removal Kit (Miltenyi Biotec) was found to be more effective for breast tissues with a 40% improvement in cell viability from the original sample, leading to its inclusion in our protocol (Figure 1C). Enrichment of viable cells using the kit can be repeated as needed to achieve the recommended yield of live cells.

Subsequently, cells were divided from the same suspension to continue with the Hi-C and scRNA-seq protocols.

A step-by-step protocol of the optimized procedure is included as Supplementary Material 1.

#### Hi-C procedure

We followed the protocol published by Díaz et al. (2018) with some modifications. Briefly, cells were brought to a volume of 437.5 μL with RPMI/10% FBS medium and cross-linked with 16% formaldehyde (Thermo Scientific) to a final concentration of 2% and incubated for 10 min at room temperature with gentle rotation. The reaction was stopped by adding 1 M ice-cold glycine (Sigma) to a final concentration of 0.125 M, incubating for 5 min at room temperature, and then for 15 min on ice. The cells were centrifuged at 400 *g*, washed with 500 μL of cold PBS, flash-frozen and stored at −70°C.

Fixed cells were thawed on ice, resuspended in 500 μL of cold cell lysis buffer (10 mM Tris-HCl pH 8, 0.2% IGEPAL CA-630, 10 mM NaCl, 1X cOmplete® protease inhibitors cocktail (Roche)), and incubated for 30 min on ice, gently mixing every 5–10 min. Subsequently, the obtained cell nuclei were pelleted at 300 x g for 10 min at 4°C and washed with 500 μL of NEBuffer r3.1 (NEB). After removing the buffer, the nuclei were suspended in 50 μL of NEBuffer r3.1 containing 0.3% SDS and incubated for 45 min at 37°C without agitation. Subsequently, 10% Triton X-100 was added to a final concentration of 1.14%, the volume was adjusted to 110 μL with water, and the samples were incubated for 45 min at 37°C with gentle rotation.

For genome digestion, 125 U of DpnII (New England Biolabs) were added in two slots. First, 50 U were added, and the sample was incubated at 37°C with gentle agitation (650 rpm) overnight. Subsequently, an additional 75 U were added, and the incubation continued under the same conditions for an additional 4 h, after which the enzyme was inactivated by heating the samples to 62°C for 20 min.

To fill in the cohesive DNA ends and mark the molecules with biotin, biotin-14-dATP (Invitrogen), and the rest of dNTPs were used together with DNA Polymerase I Klenow enzyme (New England Biolabs, 5 U/μL), and incubated for 75 min at 37°C with gentle agitation. After the incubation, DNA Ligase Buffer (New England Biolabs) was added to achieve a concentration of 1X, along with 50 U Weiss of T4 DNA Ligase enzyme (Thermo Scientific, 5 U Weiss/μL), 0.24 μg/μL Bovine Serum Albumin (BSA, New England Biolabs, 20 mg/mL), and 10% Triton X-100 for a final concentration of 0.8%, bringing the total volume to 1,000 μL.

After ligation, proteinase K (Sigma) and RNase I (Sigma) were added followed by incubation at 37°C for 2 hours and crosslinks reversed at 65°C overnight. The DNA was purified using a Phenol-Chloroform-Isoamyl alcohol mix (25:24:1) (Sigma) 1X v/v, then 0.1 volumes of 3 M sodium acetate (Sigma, pH 5.2), 50 μg/μL of glycogen (Sigma, 20 mg/mL), and 2 volumes of absolute ethanol were added. Precipitated DNA was centrifuged at 15,000 rpm for 30 min at 4°C, washed twice with 80% ice-cold ethanol, dried, and the pellet diluted in 30 μL of TLE.

Next, DNA was sheared using a Covaris sonicator, followed by a biotin pull-down using Dynabeads MyOne Streptavidin C1 beads (Invitrogen). Several washes were performed using Tween Buffer (5 mM Tris-HCl pH 8.0, 0.5 mM EDTA, 1 M NaCl, 0.05% Tween), 0.5 X Tween Buffer, and No Tween Buffer (5 mM Tris-HCl pH 8.0, 0.5 mM EDTA, 1 M NaCl). Removal of biotin molecules from non-ligated fragments was carried out by adding 15 U T4 DNA polymerase (New England Biolabs). The ends of sheared fragments were repaired with dNTPs and 5 U of DNA Polymerase I Klenow (New England Biolabs, 5 U/μL), phosphorylated with the enzyme T4 PNK (New England Biolabs, 10 U/μL), and an adenine nucleotide was added to the fragments using the enzyme DNA Polymerase I Klenow exo- (New England Biolabs). Finally, Illumina TruSeq adapters were ligated, and the libraries were sequenced on an Illumina NovaSeq - S4 flow cell.

#### Hi-C analysis

The FASTQ files originated from paired-end sequencing were processed using Seqtk (Heng, 2023) to remove low-quality regions and trim adapter sequences. The reads were aligned to the GRCh38 reference human genome using runHiC (Wang, 2016). Subsequently, using the same software, the reads were filtered for non-informative read pairs and PCR duplicates. The quality of the experiments was assessed with runHiC and HiCUP (Wingett et al., 2015). To identify SVs, HiCBreakfinder (Dixon et al., 2018) and EagleC (Wang et al., 2022b) were employed, providing both intra-chromosomal and inter-chromosomal SVs. HiNT-TL was applied for further corroboration of inter-chromosomal translocations (Wang et al., 2020). In each case, SVs were visually confirmed to eliminate false positives. The final list was constructed by combining the information provided by all the tools. After eliminating the false positive SVs, the gene fusion events were identified with EagleC, and the complex SVs reconstruction was performed using NeoLoopFinder (Wang et al., 2021).

Chromatin compartments were calculated by converting the matrices obtained through runHiC to the appropriate format and using dcHiC software (Chakraborty et al., 2022). The matrices were normalized with ICED and the analysis done at 100 kb resolution. The identification of Topologically Associating Domains (TADs) was performed using the TADLib pipeline (Wang et al., 2015; Wang et al., 2017) with the hitad command. Afterward, TADs were filtrated obtaining just the larger domains (level 0) for comparison between tumor and normal tissue matrices. TAD boundaries were compared using BEDtools (Quinlan and Hall, 2010) giving an 50 kb window to each side of the boundaries. Matrices were visualized using HiGlass (Kerpedjiev et al., 2018).

For normal tissue, two published *in situ* Hi-C experiments (Kim et al., 2022) were merged to achieve a valid pair count like the Hi-C of the primary breast cancer tumor. To compare the SVs detected in our TNBC tumor, we analyzed three published *in situ* Hi-C experiments from TNBC tumors (Kim et al., 2022) and called SVs, as described in the sections above.

### scRNA-seq procedure

The scRNA-seq libraries were constructed using the Chromium Next GEM Single Cell 3′Reagent Kits v3.1 (Dual Index) following the manufacturer’s instructions (10X Genomics). Briefly, the cell suspension was minimally manipulated as described above and loaded onto the Chromium Next GEM Chip G, avoiding bubble formation. Then the chip was inserted into the Chromium Single Cell Controller. Subsequently, the cDNA library was produced, cleaned, and amplified using 11 cycles of the specified PCR program. Libraries were constructed by adding the appropriate adapters and indices (Dual Index TT Set A) provided by the manufacturer. The final amplification was carried out with 12 cycles of the specified PCR program and sequenced on an Illumina NovaSeq - S4 flow cell.

### scRNA-seq analysis

#### Quality metrics and clustering analysis

FASTQ reads obtained from the pair-end sequencing were aligned to the GRCh38 reference human genome and quantified using Cell Ranger (version 3.1, 10X Genomics). For quality control, standard filters were used, and cells with >15% of mitochondrial UMI´s (Unique Molecular Identifier) were excluded from analysis (Zhang et al., 2021a; Tietscher et al., 2023). Genes detected in fewer than 3 cells and within cells with less than 200 genes were filtered out.

After quality control, 5, 591 cells were obtained in total, 5,201 cells from the tumor and 390 cells from the adjacent tissue. The “integration” function and the batch correction were performed using the CCAIntegration method with default parameters. Library normalization, scaling of the data, dimensional reduction, clustering and the previous steps were performed with the Seurat package (Stuart et al., 2019). The elbow plot and the number of principal components were obtained with the ElbowPlot function. In this case, the 20 first principal components were used. Evaluation of the cell cycle was calculated using the CellCycleScoring function. Visualization of clusters was performed with UMAP (Uniform Manifold Approximation and Projection). The function FindAllMarkers was utilized to identify genes that characterize the clusters and identification of cell populations was made using canonical markers: *PTPRC* (*CD45* ^+^) for immune cells; *MKI67* for proliferative cells; *CD3D* for NKT/T cells; *MS4A1* for B cells; *CD68* for myeloid cells; *PECAM1* for endothelial cells; *EPCAM* for epithelial cells; *PDGFRB* for mesenchymal cells and *JCHAIN* for plasmablasts. Also, additional markers were used to identify subpopulations like fibroblasts with the expression of *TNC, COL18A1* and *COL12A1* (Wu et al., 2021a; Kumar et al., 2023; Tietscher et al., 2023). Additionally, within the T cell population, a subpopulation of regulatory T cells was detected, through the expression of *CTLA4* and *FOXP3*.

For the PAM50 cluster map analysis, the list of the 50 gene markers was obtained from the original paper (Parker et al., 2009). The counts of these gene transcripts were selected in all tumor and adjacent tissue cells and the hierarchical clustering was calculated using Euclidean distance and Ward linkage method.

The data normalization and cell population separation were done using R and Seurat (Hao et al., 2024) library. The statistical analysis and cluster map were performed using Python *ad hoc* scripts with Scipy (Virtanen et al., 2020), Pingouin (Vallat, 2018), and Seaborn (Hunter, 2007; Waskom, 2021) libraries.

## Results

### Successful obtention of viable cell suspensions

To successfully process primary breast cancer tumor biopsies, a crucial first step was standardizing sample handling and storage to have a functional protocol that allows sample storage while maintaining cell viability for further processing as clinical biopsies may be obtained and delivered to the laboratory at unpredictable times.

Two protocols for sample handling and storage were tested: one involving the freezing of cell suspensions and the other storing the sample as frozen tissue. Both protocols were tested using tissue obtained from primary breast cancer tumors (n = 6 for frozen cell suspensions and n = 24 frozen tissues).

Using the pre-freeze dissociation protocol the average viability of the 6 samples processed was 34.78% and only 1 passed viability QC (>70%) upon thawing, making up 16.6% of the samples (Figure 1D). Out of the 5 remaining samples, we were able to rescue only 2 with the dead cell removal kit, representing 40% of the samples (Figure 1D).

In contrast, 8 samples out of 24, or 33.3% of the samples, were viable when processed with the post-freeze protocol proposed. After using the dead cell removal kit on 16 of the remaining samples, we rescued 12, representing 75% of the group. On average, using this method, 20 out of 24 samples (83.3%) passed the quality control to continue with the protocol (Figure 1D).

More importantly, the total number of recovered cells was higher from the frozen tissue storage protocol, due to the elimination of handling steps (Figures 1A,B). Considering these results, we proceeded with the frozen tissue storage protocol.

With the handling, storage, and disaggregation protocol established, a primary Triple Negative Breast Cancer tumor sample (TNBC) was processed. The cell suspension obtained for this sample consisted of approximately 1.4 million cells, exhibiting 92% cell viability. This cell suspension was divided into two parts, one for Hi-C and one for scRNA-seq. For the adjacent tissue, we recovered ≈1100 cells used for scRNA-seq.

### High-quality Hi-C and single-cell RNA-seq data sets from primary breast cancer biopsies

**FIGURE 2:**
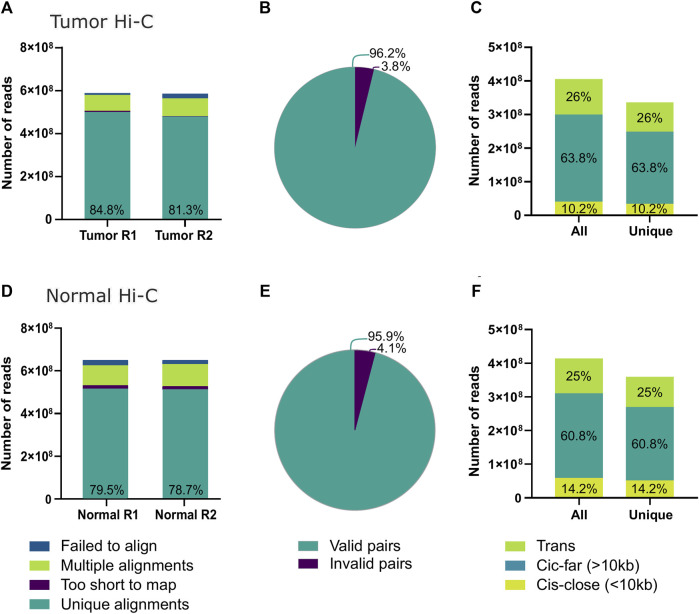
Hi-C quality from the tumor biopsy using the post-freeze tissue dissociation protocol. **(A)** Number of successfully aligned sequencing reads from the tumor Hi-C to the GRCh38 reference genome. **(B)** Number of Hi-C reads identified as informative contacts for interaction matrix construction; within the category of invalid pairs wrong size sequences, circularized reads, non-ligated, re-ligated, and continuous sequences are included. **(C)** Total and unique reads from tumor Hi-C counts after PCR duplicate removal. Additionally, the percentages of contacts identified within less than 10 kb distance (Cis-close), more than 10 kb distance (Cis-far), or between different chromosomes (Trans) are shown. **(D)** Number of successfully aligned sequencing reads from the normal tissue Hi-C to the GRCh38 reference genome. **(E)** Number of normal tissue Hi-C reads identified as informative contacts for interaction matrix construction. **(F)** Total and unique reads from normal tissue Hi-C counts after PCR duplicate removal divided by contact distance.

Approximately 1.3 million tumor cells were destined for Hi-C. The experiment’s quality was assessed using the HiCUP software (Wingett et al., 2015) (Figure 2; Supplementary Table 1). Nearly 80% of the sequenced read pairs aligned to the reference genome uniquely (Figure 2A). Experimental artifacts, such as non-ligated or circularized regions, accounted for approximately 4% of the sequencing read pairs, indicating a high execution efficiency and high-quality production of interaction data sets (Figure 2B). Finally, 17.07% of the reads were identified as PCR duplicates leaving a total of 336 million valid paired reads (Figure 2C).

The percentage of contacts in cis-close (<10Kbp), cis-far (>10Kbp), and trans was 26%, 63.8%, and 10.2%, respectively (Figure 2C). Since the probability of contacts decreases with genomic distance, the proportion of intrachromosomal contacts is typically three times higher than that found between chromosomes when using the Hi-C technique (Lieberman-Aiden et al., 2009; Sarnataro et al., 2017). For this reason, the experiment met the quality standards for the analysis and construction of interaction matrices.

Additionally, quality was also assessed for the published Hi-C data sets from healthy breast tissues (Figures 2D–F) and for the additional published Hi-C data from TNBC tumors analyzed. All data sets presented similar quality control measures, good enough to continue with further analysis (Supplementary Table 1).

For the tumor scRNA-seq, 16,000 cells were allocated with an approximate viability of 92%, calculated using Trypan Blue. Considering an approximate loss of 60% inherent to the single-cell capture technique, we would expect about 8,832 viable cells. For the adjacent tissue approximately 1,100 viable cells were processed.

A preliminary count of 6,264 cells was obtained from the scRNA-seq data, an average of 66,329 reads, and a median of 3,260 genes per cell was obtained in the tumor sample. For the adjacent tissue, 481 cells, 61,593 reads and 1,195 genes per cell were obtained, indicating a good result according to the manufacturer’s specifications (10X Genomics).

The Barcode Rank Plot shows an adequate number of UMIs associated with barcodes, which confirms a correct cell capture. Furthermore, few cells showed a high percentage of mitochondrial transcripts, those cells were filtered out (Supplementary Figure 1A) and after quality control we obtained cells with appropriate RNA molecules, features (genes) and mitochondrial counts (Supplementary Figure 1E), thus it was considered that the experiment exhibited adequate quality for further analysis (Chen et al., 2019; Luecken and Theis, 2019). Additionally, the distribution of cells in different phases of the cell cycle was also identified and it showed a distribution consistent with the cell heterogeneity in a tumoral sample (Supplementary Figures 1B, D). Therefore, out of a total of 6,264 cells for the tumor and 481 cells for the adjacent tissue, there was a reduction of 11%, resulting in 5,591 (5,201 cells for the tumor and 390 for the adjacent tissue) viable cells for subsequent analyses after quality control. These results confirm the success of the scRNA-seq experiment (Supplementary Table 2).

The single-cell RNAseq data sets presented here represent two examples of technically successful experiments with different cell counts derived from breast biopsies. In the literature, most single-cell RNAseq data sets with variable cell counts are analyzed by pooling samples from many patients or conditions to increase the cell numbers considered in the analysis (Qian et al., 2020; Gao et al., 2021; Pal et al., 2021; Reed et al., 2024). Thus, the observations made here are just a description of the different cell populations found in these two data sets with all their limitations. More complex samples and analyses are needed to confirm and expand the observations presented more comprehensively.

### Identification of cell populations in the TNBC tumor

**FIGURE 3:**
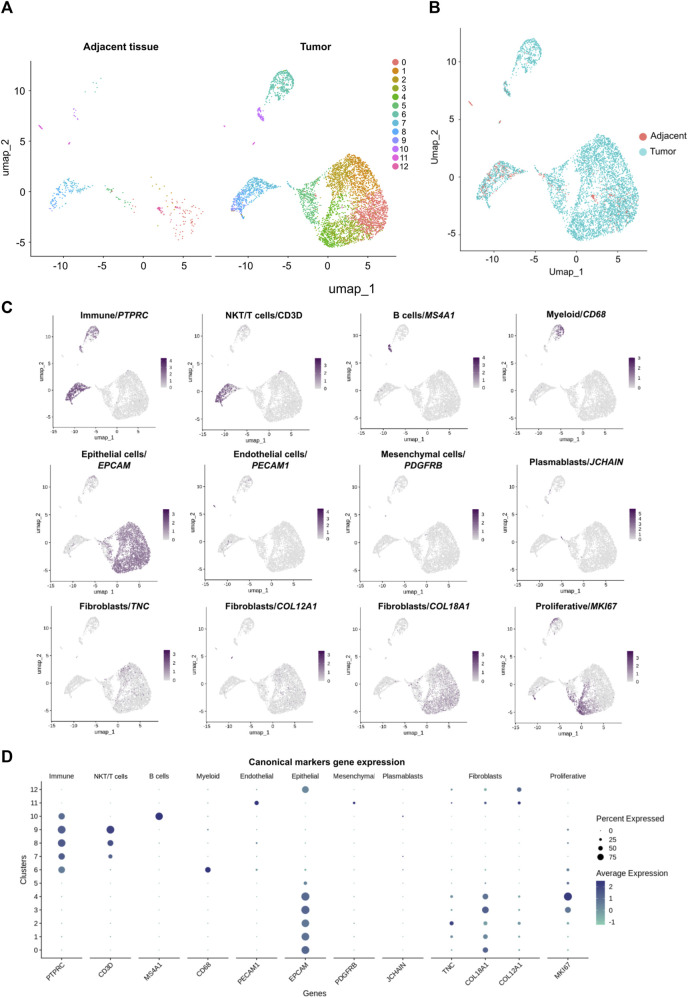
Characterization of cell populations derived from the breast cancer tumor and the adjacent control tissue scRNA-seq data sets. **(A)** UMAP of the 390 cells from the adjacent tissue and UMAP of the 5,201 cells from the tumor colored by cell the cell clusters detected (0–12). **(B)** UMAP of 5,591 cells: 5,201 cells of tumor (blue cells) and 390 cells of adjacent tissue (red cells). **(C)** Identification of subpopulations with canonical markers and their expression across the clusters: *PTPRC* (*CD45* ^*+*^) for immune cells; *MKI67* for proliferative cells; *CD3D* for NKT/T cells; *MS4A1* for B cells; *CD68* for myeloid cells; *PECAM1* for endothelial cells; *EPCAM* for epithelial cells; *PDGFRB* for mesenchymal cells and *JCHAIN* for plasmablasts. Also, additional markers were used to identify a particular subpopulation like fibroblasts with the expression of *TNC*, *COL18A1* and *COL12A1*. **(D)** Canonical markers expression across the clusters.

Characterization of the immune subpopulations infiltrating the tumor by scRNAseq has been a useful tool to predict tumor control and immunotherapy responsiveness (Azizi et al., 2018; Sade-Feldman et al., 2018; Li et al., 2019); hence we aimed to define the identity of different tumor-infiltrating immune cell populations including T cells, B cells and myeloid cells. Our protocol allowed the identification of eight clusters of non-hematopoietic cell populations based on the negative expression of *PTPRC* (*CD45* ^*−*^ *)* and the heterogenous expression of *EPCAM* gene transcripts defining epithelial cells (Figures 3C,D). Also, we identified the expression of endothelial (*PECAM1*), plasmablasts (*JCHAIN*) and mesenchymal (*PDGFRB*) cell transcript markers according to Wu et al., 2021a (Figures 3C,D). Finally, the expression of *TNC*, *COL18A1* and *COL12A1* gene transcripts related to fibroblast subtypes was also detected (Tan et al., 2022) (Figures 3C,D). There were cells from both adjacent tissue and tumor samples contributing to most clusters (Figures 3A,B) and the most abundant cluster in both tissues is cluster 0. Interestingly, clusters 3 and 4 contain 1 and 0 cells from the adjacent tissue respectively, and these represent the most proliferative cells from the epithelial compartment in the tumor (Figures 3C,D; Supplementary Figures 1C, D. On the contrary, endothelial and mesenchymal gene transcript markers were primarily detected in clusters 11 and 12 which are enriched with cells from the adjacent tissue with 10 and 3 cells from the tumor respectively (Figures 3A,C,D).

Each cluster was characterized by an independent transcriptional profile, in addition to the identification of canonical markers (Supplementary Figure 1F). Two groups of cell populations were identified: hematopoietic cells expressing *PTPRC* (*CD45* ^*+*^) were included in clusters 6 to 10; non-hematopoietic cells (without expression of *PTPRC*) comprised clusters 0 to 5, 11, and 12. Each cluster of hematopoietic cells expressed specific genes. Cluster 6 was identified as myeloid cells with the expression of *CD68* as a canonical marker, and transcriptomic signature including *TYROBP*, *AIF1*, *FCER1G*, *IFI30*, *LYZ*, *LAPTM5*, *PLEK*, *SPI1*, *ITGB2*, *LST1.* Clusters 7, 8 and 9 belonged to T/NKT cells with expression of *CD3D*, *GZMA*, *GZMB GZMH* and *CD8A* genes. Cluster 9 represented a subtype of T cells, known as regulatory T cells, distinguished by *FOXP3* gene expression. These regulatory T cells also express *CTLA4*, *ICOS*, *LAIR2* and *TIGIT* gene transcripts. Finally, cluster 10 was identified as B cells expressing *MS4A1* and *CD79A* genes. For the non-hematopoietic component, all clusters expressed *EPCAM* gene, a marker for epithelial cells, but every cluster had a particular transcriptional signature. Cluster 0 to 5 have similar genes expressed but at different levels. For example, clusters 1 and 2, expressed *VCAM1*, *LTF*, *ITGB8* and *CXCL1* while clusters 0, 3 and 4 expressed less of these transcripts. Clusters 0, 3 and 4 shared some gene transcripts such as *GABRP*, *ITPR2*, *NOTCH3* and *VTCN1*, but with low levels in clusters 3 and 4. Clusters 3 and 4 differed from cluster 0 as they expressed *RAD51AP1*, *TK1*, *GINS2*, *HELLS*, *CENPU*, *CLSPN*, *TPX2*, *PRC1*, *CENPE* and *CEPF* genes which are not expressed in cluster 0. Cluster 12, which also expressed the *EPCAM* gene, expressed unique genes including *LIN7A*, *CAPN8*, *CLASTN2* and *TTC6*. On the other hand, cluster 11 did not express *EPCAM* but expressed the endothelial marker gene *PECAM1*. The cells in this cluster also expressed *TIMP3*, *CXCL12*, *CLDN5*, *CD34*, *EMCN* and *CDH5* genes.

Within the five clusters expressing *EPCAM* epithelial marker together with *COL18A1* (clusters 0–4), two presented a high number of cells in the S and G2M phases of the cell cycle and are enriched with tumoral cells. These populations were identified as proliferative epithelial cells by the expression of *MKI67* (Figures 3C,D; Supplementary Figures 1C, D) which might represent the tumoral epithelial cell compartment with a proliferative transcriptional program.

Additionally, we were able to classify the primary breast cancer tumor by using PAM50 markers on our scRNA-seq data (Supplementary Figure 2). We observed several basal-like marker genes expressed in the tumor sample and the lack of *ERBB2* amplification and hormonal receptors. These expression profile in the tumor sample of the PAM50 marker genes allows us to suggest that our TNBC tumor was possibly of the basal-like subtype (Perou et al., 2000; Wallden et al., 2015).

With these results we concluded that the processing and storage of the primary breast cancer tumor biopsy sample was successful for the implementation of Hi-C and scRNA-seq experiments.

### Characterization of SVs in the TNBC tumor

Interaction matrices were constructed using the runHiC software (Wang, 2016). Published *in situ* Hi-C data set from normal breast tissue was used as a healthy reference and three Hi-C data obtained from TNBC tumors were used to compare the SVs (Kim et al., 2022).

**FIGURE 4:**
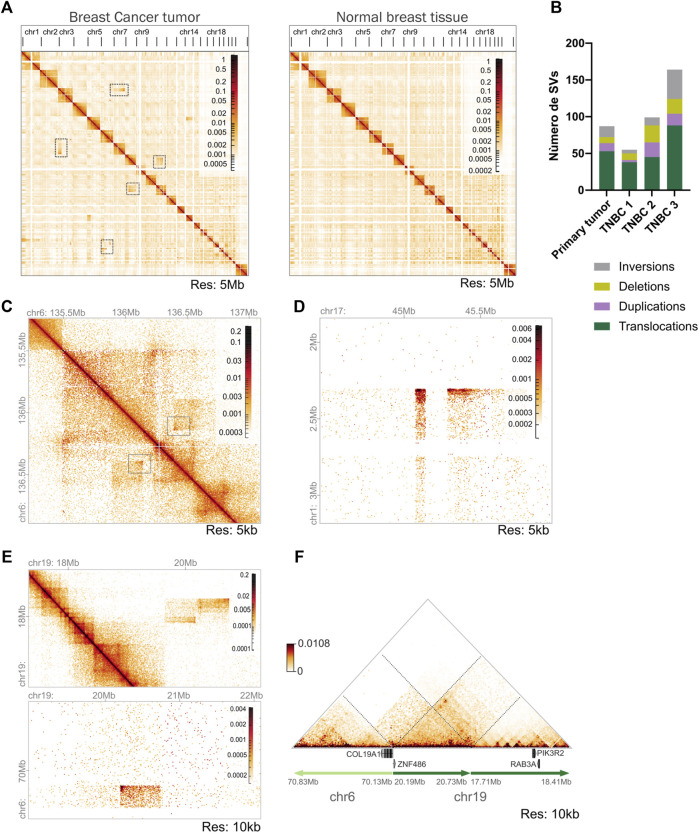
Identification of SVs in the triple negative breast (TNBC) tumor using Hi-C data. **(A)** Whole-genome interaction matrices of the tumor tissue biopsy sample and previously published normal breast tissue. It is observed that the highest frequency of contacts is in *cis* in both samples. High-frequency interchromosomal interactions observed in tumor tissue and not in normal breast tissue are highlighted with boxes. Matrices constructed at 5 Mb resolution. **(B)** Number and classification of SVs found in the Hi-C tumoral sample processed and compared with the TNBC Hi-C data published by Kim et al. (2022). **(C)** Hi-C contact matrix showing a deletion in chromosome 6 identified by the SVs analysis. Resolution 5 kb. **(D)** Inter-chromosomal Hi-C matrix showing a translocation between chromosomes 1–17. Resolution 5 kb. **(E)** Intra and inter-chromosomal Hi-C contact matrices showing a complex SV formed involving a translocation between chromosomes 6–19 and a duplication in chromosome 19. **(F)** Reconstruction of the loci altered by a complex SV. Translocation between chromosomes 6–19 generates a gene fusion of *COL191A* and *ZNF486* genes identified by EagleC analysis. Duplication in chromosome 19 harbors genes possibly related to tumoral activity.

Whole-genome interaction matrices were constructed, revealing a similar cis/trans interaction trend in our tumor and normal datasets. However, several high-frequency interchromosomal interactions were detected in the tumoral genome which were absent in the normal breast tissue matrix identified as potential chromosomal translocations (Figure 4A).

Next, we analyzed the tumor to detect SVs. Using three different identification software packages, a total of 87 SVs were found, which were visually confirmed in the interaction matrices. Among these, 53 corresponded to inter-chromosomal translocations and 34 to intra-chromosomal SVs encompassing 15 inversions, 11 duplications, and 8 deletions of genetic material (Figure 4B).

TNBC is known for its heterogeneous and unstable genome (Kwei et al., 2010), especially the basal-like subtype. To compare the SVs identified in our tumor we evaluated three published Hi-C data sets from TNBC-diagnosed female patients (Kim et al., 2022). We observed a high genomic instability in all the samples processed accompanied by elevated heterogeneity between the samples. The TNBC3 sample was the most rearranged with 164 SVs in total, followed by TNBC2 with 99 SVs our tumor with 87, and finally TNBC1 with 55 SVs (Figure 4B). These findings recapitulate the previous observations that TNBC tumors present a variable number of SVs and high heterogeneity between individual samples (Supplementary Figure 3A) (Kawazu et al., 2017).

Next, we compared the regions affected by SVs in the published TNBC published samples in contrast to our tumor. We found 6 regions of intra-chromosomal SVs shared between our sample and TNBC1. For the TNBC2 we found 25 regions were involved in genomic alterations in both samples, especially in chromosome 6 (Supplementary Table 3). These regions encompassed 242 genes, including some ones previously related with oncogene activity in TNBC breast cancer like *NOTCH3* and *PKN1* (Supplementary Figures 3B, C) (Turner et al., 2010; Kawazu et al., 2017; Kovalevska et al., 2021). Finally, the comparison made with the TNBC3 sample showed less similarity accounting for only two SVs with shared genomic regions. This analysis indicates that our primary TNBC tumor more closely resembles the TNBC2 sample in terms of SVs.

Using our Hi-C data we also analyzed the resulting gene fusions generated by the SVs previously identified. We found 19 SVs involving 38 genes in gene fusion events in the primary tumor sample (Supplementary Table 4). From these genes, 19 were found to be significantly expressed in the scRNAseq data. The same analysis was performed on the published TNBC Hi-C matrices, from which 18 SVs were found to generate gene fusion events in TNBC 1, 26 SVs in TNBC 2 and 31 in TNBC 3 (Supplementary Table 4). Nevertheless, none of the genes found fused in the primary tumor sample was shared with any of the published data indicating that TNBC subtype molecular alterations are highly heterogeneous between patients.

An example of an SV identified in our tumor in contrast with the normal tissue is a deletion pattern in chromosome 6 of 250 kb length in the Hi-C matrix (Figure 4C). From this deletion a gene fusion was identified involving *PDE7B-MAP7* genes. *MAP7* gene has been found to promote cell migration and invasion in breast cancer tumors (Wang et al., 2022a; Wang et al., 2023b) and *PDE7B* gene encodes a phosphodiesterase that is involved in the regulation of cellular cAMP.

The deletion also encompassed *MTFR2* and *BCLAF1* genes, the latter one was recently related to the regulation of the PDL1 pathway in breast cancer (Ma et al., 2021). Both gene overexpression is related to a worse prognosis in breast cancer (Lu et al., 2020; Yu et al., 2022).

Another SV found in the tumor sample was an inter-chromosomal translocation between chromosomes 1–17 (Figure 4D). This translocation was found to generate a gene fusion between *SKI*-NMT1 genes, *SKI* is an interesting gene reported both with a pro-oncogenic and a suppressor tumor activity in breast cancer models (Rashidian et al., 2015). The *NMT1* gene was highly expressed in the tumor and is reported as a potential diagnostic biomarker in breast cancer related to poor prognosis (Wang H. et al., 2023).

Our Hi-C data also allowed the identification of complex rearrangements involving more than one SV in the same locus (Figures 4E,F), one of which encompasses a duplication found in chromosome 19 (coordinates: 17,710,000–20,735,000) and a translocation of chromosomes 6–19 (coordinates: chr6:70,130,000 chr19:20,195,000). The complex SV was reconstructed using NeoLoopFinder and was found to generate *COL19A1*-*ZNF486* gene fusion. COL19A1 is an alpha chain of type XIX collagen found to be related to immunotherapy response in esophageal squamous cell carcinoma (Liu J. et al., 2023). *ZNF486* codes for a zinc-finger protein that has been described as a potential prognosis marker in breast cancer patients (Du et al., 2021), however more data would be needed to characterize its specific function in breast cancer cells. The duplicated region also harbored *PIK3R2* and *RAB3A* genes, both of which have a role in several malignancies (Wu et al., 2018; Liu et al., 2022). Both genes are being expressed in the tumor (Figures 4E,F).

**FIGURE 5:**
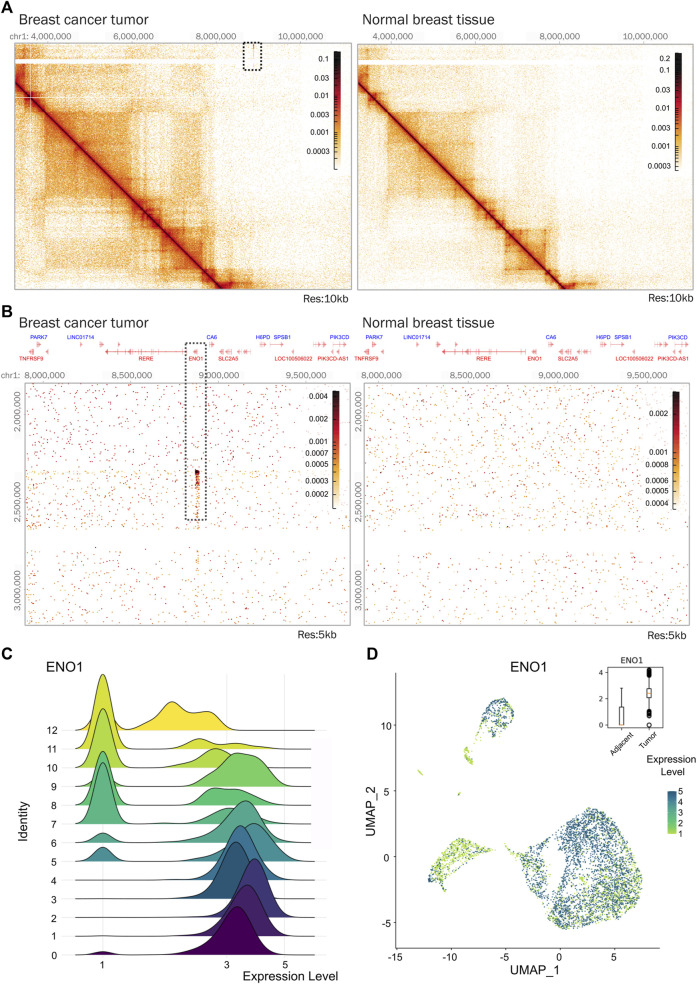
*ENO1* gene is amplified in the triple negative breast tumor. **(A)** Hi-C contact matrices comparing the region where gene *ENO1* is located in tumor tissue and normal breast tissue. The area showing an interaction indicating amplification of genetic material in tumor tissue is marked with a square. Matrices at 10 kb resolution. **(B)** Zoom-in on the region showing an amplification pattern that co-localizes with the *ENO1* gene locus in tumor tissue. Matrices at 5 kb resolution. **(C)** Distribution of *ENO1* expression in tumor and adjacent tissue cells of breast cancer. **(D)** Density expression of *ENO1* across the clusters. Expression is plotted on the box plot.

Another example of a genomic region that was altered in our tumor, corresponds to that encoding the α-enolase (*ENO1*) gene. This region was duplicated and involved in a gene fusion. *ENO1* gene expression is increased in various cancer types, including breast cancer (Cancemi et al., 2019) (Figures 5A,B). The α-enolase is involved in glycolysis and has shown oncogenic properties related to promoting cell proliferation and tumor metastasis (Xu et al., 2020; Huang et al., 2022). Silencing of *ENO1* gene in a breast cancer cell line reduced the proliferative capacity of the cells (Zhang et al., 2020), indicating that *ENO1* could be a potential molecular prognostic marker or therapeutic target in breast cancer.

In our samples, *ENO1* was significantly expressed in the tumor (Figure 5D) and expressed at different levels the cell clusters identified, particularly in epithelial populations enriched in the tumor sample (Figures 5C,D). This could suggest that the epithelial cell populations found in the tumor could have different metabolic cell states reflected by *ENO1* gene expression levels. Although further analysis is necessary, the presence of a genetic duplication in the tumor could potentially be a driving factor altering its expression. The application of Hi-C in more samples could confirm if this duplication is a common feature in breast cancer and functional experiments would be needed to determine causality.

### Identification of chromatin compartments and topologically associating domains in the TNBC tumor

**FIGURE 6:**
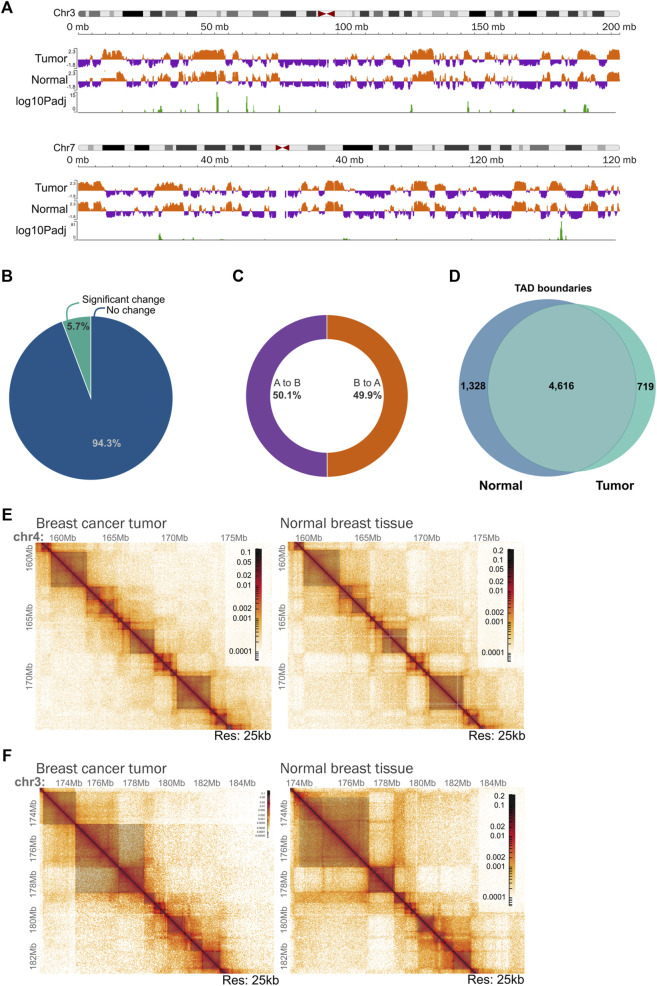
Chromatin organization of the triple negative breast tumoral genome at different scales. **(A)** Example of chromatin compartmentalization of two chromosomes (chr3 and chr7) between tumoral and normal tissue. **(B)** Percentage of the genome that changes significantly from compartment in the tumor in comparison with normal tissue. **(C)** Percentage of the changing chromatin that goes from A to B and B to A compartment in the tumor in comparison with normal tissue. **(D)** Conservation of TAD boundaries across the analyzed samples. TAD boundaries were calculated using TADlib. **(E)** Example of a region on chromosome 4 showing a very similar organization of Topologically Associating Domains (TADs) between tumor tissue and normal tissue. Dark gray squares represent TADs identified using TADlib. Matrices at 25 kb resolution. **(F)** Example of a region on chromosome 3 showing different TADs and compartmentalization organization between tumor tissue and normal tissue. Dark gray squares represent TADs identified using TADlib. Matrices at 25 kb resolution.

Next, we explored the overall genome topology of the tumor in contrast to the normal breast tissue. The interaction patterns corresponding to A and B chromatin compartments exhibited few differences as in most regions the pattern was similar between the two samples (Figures 6A,B; Supplementary Figure 4). The percentage of the genome-changing compartment was low representing less than 6% (Figure 6B). From these regions, nearly half changed from A to B, and the other 50% presented a pattern change from B to A compartment (Figure 6C). Even if the changes at the compartment level are small, further analysis is needed to characterize if gene expression changes could occur derived from compartment switching.

Regarding Topologically Associating Domains (TADs), 5,927 TADs were found in the normal tissue and 5,212 in the tumor sample with an average size of 470 kb and 540 kb respectively. We noticed that the structures are highly conserved between the two samples with 70% of the boundaries shared between them (Figures 6D,E). There were places in which clear differences were detected (Figure 6F), indicating that the Hi-C experiment successfully provided the tumoral genome topology at different scales. To thoroughly investigate the functional consequences of the differences in TAD boundaries between the tumoral genome and the normal breast tissue, additional analysis is needed.

## Discussion

We have presented an optimized and cost-effective protocol to store and process breast cancer tumor samples acquired through core needle biopsies to obtain viable and debris-free single-cell suspensions. The quantity, viability and quality of cell suspensions are determining factors that limit the potential use of this sample type in various experimental protocols and each type of tissue and cellular composition presents its challenges. Many strategies have been proposed to work with biopsies from different sources (Díaz et al., 2018; Slyper et al., 2020; Wu et al., 2021b; Burja et al., 2022). The alternative described here is an affordable option yielding high-quality cell suspensions for scRNA-seq and Hi-C experiments.

For Hi-C and other chromosomal conformation capture techniques the samples can be directly fixed with formaldehyde and then frozen (Díaz et al., 2018). However, this is not the case for experiments in which cells need to be viable as for scRNA-seq. Processing fresh tissue for scRNA-seq can yield good results (Slyper et al., 2020). However, when working with tissue derived from clinical procedures, it is not always feasible to experiment on fresh samples. The protocol presented here enables the acquisition of high-quality data, both transcriptomic and topological, allowing the characterization of different levels of molecular dysregulation in tumor biopsies.

The molecular characterization of SVs using Hi-C has its limitations as only large SVs can be identified in the tumor sample and other types of genomic alterations such as small rearrangements or punctual mutations cannot be identified with this technique. However, Hi-C allows efficient identification of large rearrangements *de novo* and is the technology that can inform also on the topological consequences of the SVs and at the same time, topological alterations that are not derived from genetic events.

As an example of the power of the combination of the two data sets produced, we found a genetic duplication of the *ENO1* gene locus in the tumor (Figure 5B). Also, the *ENO1* gene appears expressed in most epithelial tumoral cell populations identified through scRNA-seq from the biopsies (Figure 5D). ENO1 is involved in glycolysis and its gene expression has been reported to be dysregulated in various cancer types (Huang et al., 2022). Although further analysis and functional experiments are required, together these observations suggest that the genetic duplication of *ENO1* could lead to its expression in the epithelial populations from the tumor and this may impact their metabolism. We also detected other SVs harboring genes that were expressed in the tumor as the region encompassing *NOTCH3* and *PKN1* genes and many other genes involved in gene fusions in the tumoral genome.

From the single-cell transcriptomic analysis, we found that the expression of canonical marker genes delimited the clusters of the immune component including T cells, B cells and Myeloid cell transcript markers, and a more hybrid scenario was found for the epithelial and fibroblast cell populations in the tumor (Wu et al., 2021a). The expression of the epithelial transcript marker *EPCAM* coincided with the expression of *COL18A1*, *TNC* and *COL12A1* gene transcripts in the tumor clusters (Figures 3A–C). *TNC*, *COL18A1* and *COL12A1* gene expression characterize fibroblasts known as ECM-CAF (Extracellular Matrix-Cancer Associated Fibroblasts) which participates in the aberrant remodeling of the extracellular matrix in breast cancer (Papanicolaou et al., 2022; Minini and Fouassier, 2023). Our tumor may have an overrepresentation of cells that co-express both *EPCAM* and CAF transcript markers which might represent epithelial cells transiting to a more “mesenchymal/fibroblast” transcriptomic phenotype. Moreover, these same clusters present a high expression of *ENO1*, and thus might have a more non-oxidative metabolism. Further analysis and functional experiments will be needed to expand these observations.

*PDGFRB*, *JCHAIN* and *PECAM1* are markers that could identify mesenchymal, plasmablasts and endothelial cells, respectively (Wu et al., 2021a). We observed a low expression of these markers in our tumor and their accumulation in cell populations from the adjacent control tissue. This reduced expression could be the result of the low cell number of these populations in our tumor as not all breast cancer tumors present equal proportions of these cell populations (Wu et al., 2021a).

Performing other types of analyses like Gen Set Enrichment Analysis (GSEA) or Gen Regulatory Network Analysis that could delve further into the identification of a more unconventional stromal component that is not evident using conventional markers, could add valuable information (Fan et al., 2022).

In conclusion, the possibility to conduct both 3D genomic reconstructions and single-cell transcriptomics on the same sample offers numerous opportunities to characterize in detail the genomic events that lead to genome topology aberrations and transcriptional deregulation in particular cell populations from the same tumor. This combined strategy could help to identify prognostic or therapeutic targets that have consequences on gene transcription regulation as well as the different cell populations that constitute the tumor.

## Data Availability

The original contributions presented in the study are publicly available. This data can be found here: GEO, accession number GSE270363. Publicly available datasets were also analyzed in this study. This data can be found here: GEO, accession numbers GSM5098076 and GSM5098078 (normal tissue) and GSM5098079, GSM5098080, and GSM5098082 (TNBC tumors).
