# Supplementary figures and images for: Obtention of viable cell suspensions from breast cancer tumor biopsies for 3D chromatin conformation and single-cell transcriptome analysis
Source: Front Mol Biosci. 2024 Aug 22;11:1420308. doi: 10.3389/fmolb.2024.1420308 (PMC11375512; doi:10.3389/fmolb.2024.1420308)

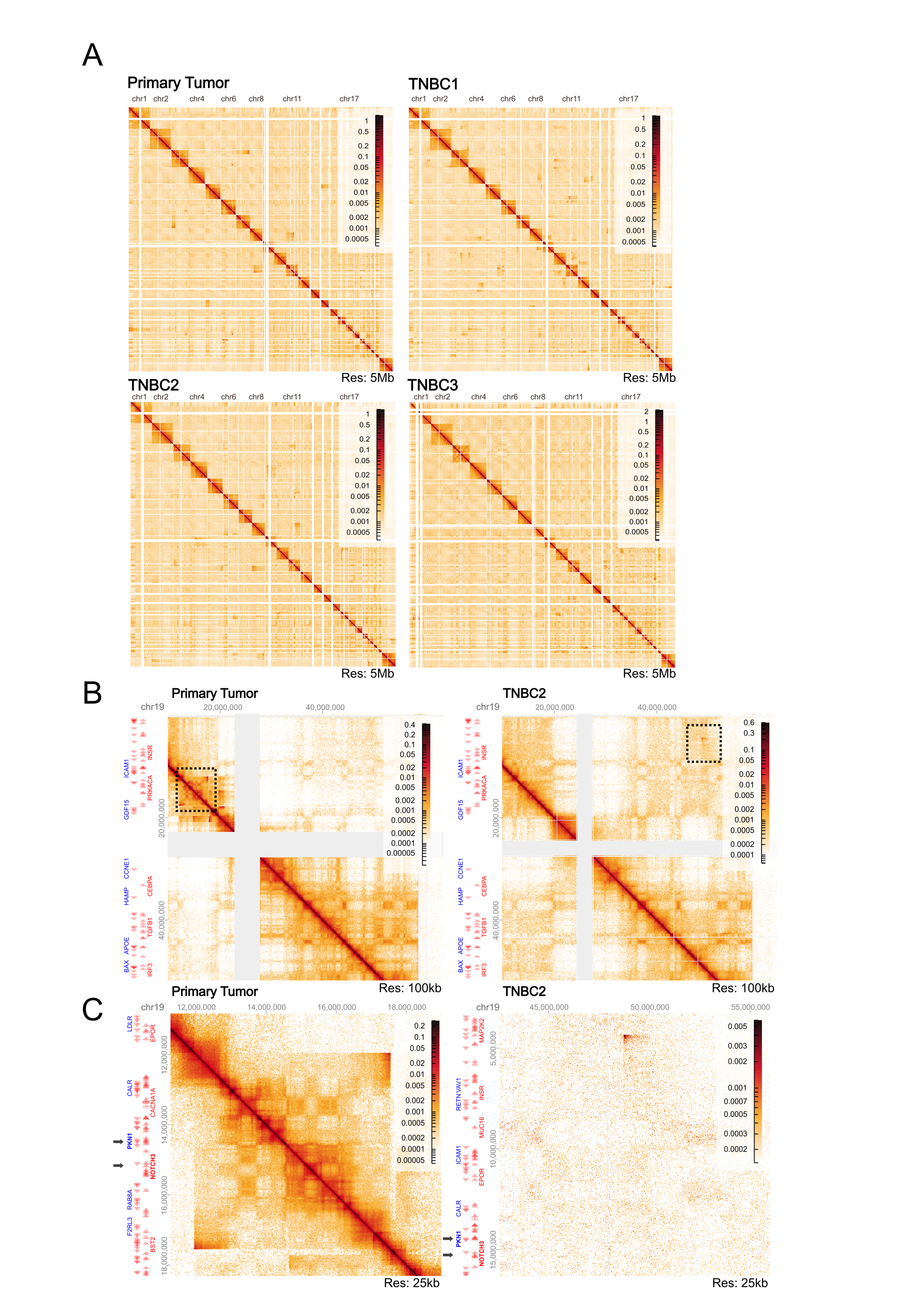

Supplement: Supplementary file 1 [file Image3.TIFF]

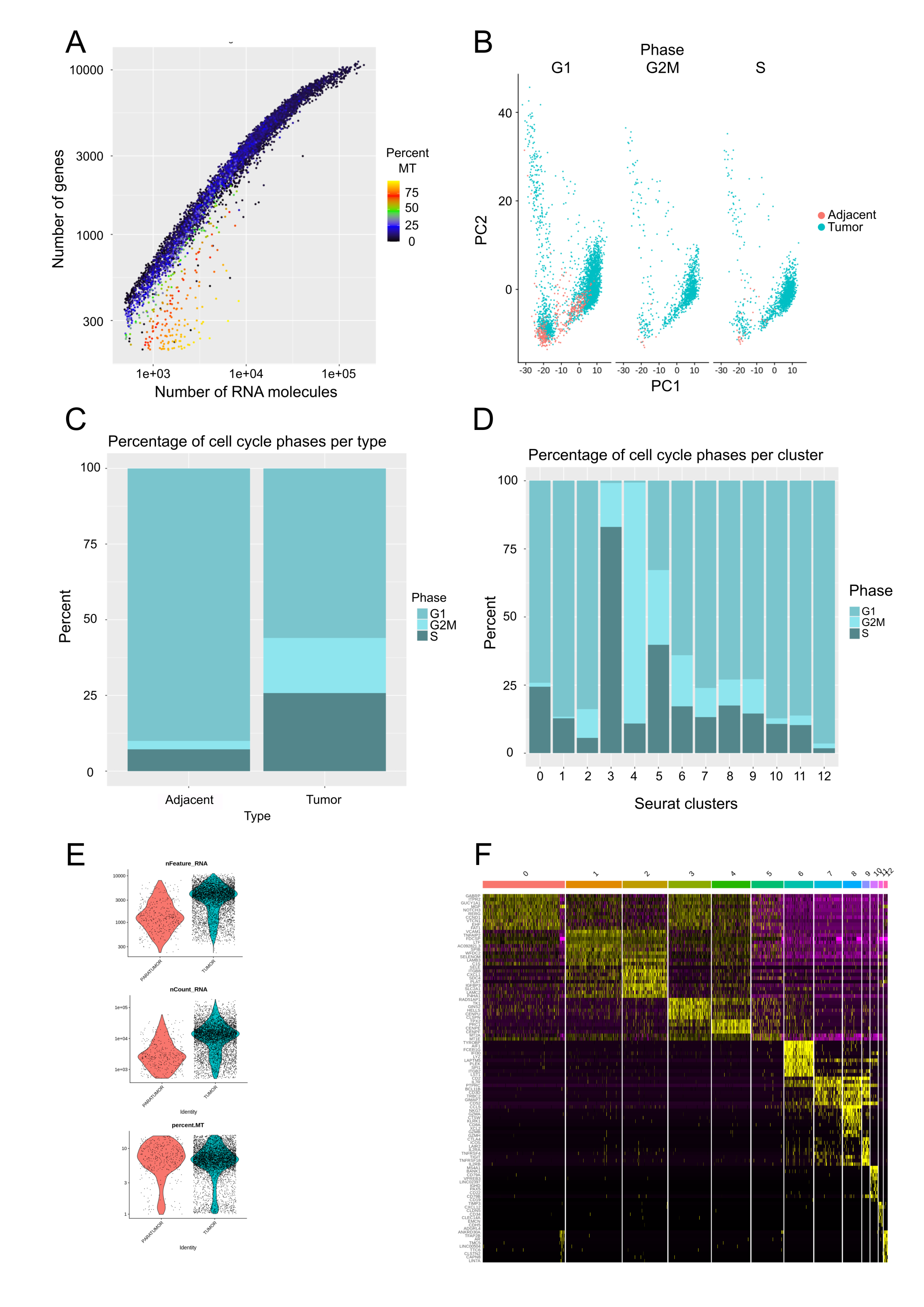

Supplement: Supplementary file 3 [file Image1.TIFF]

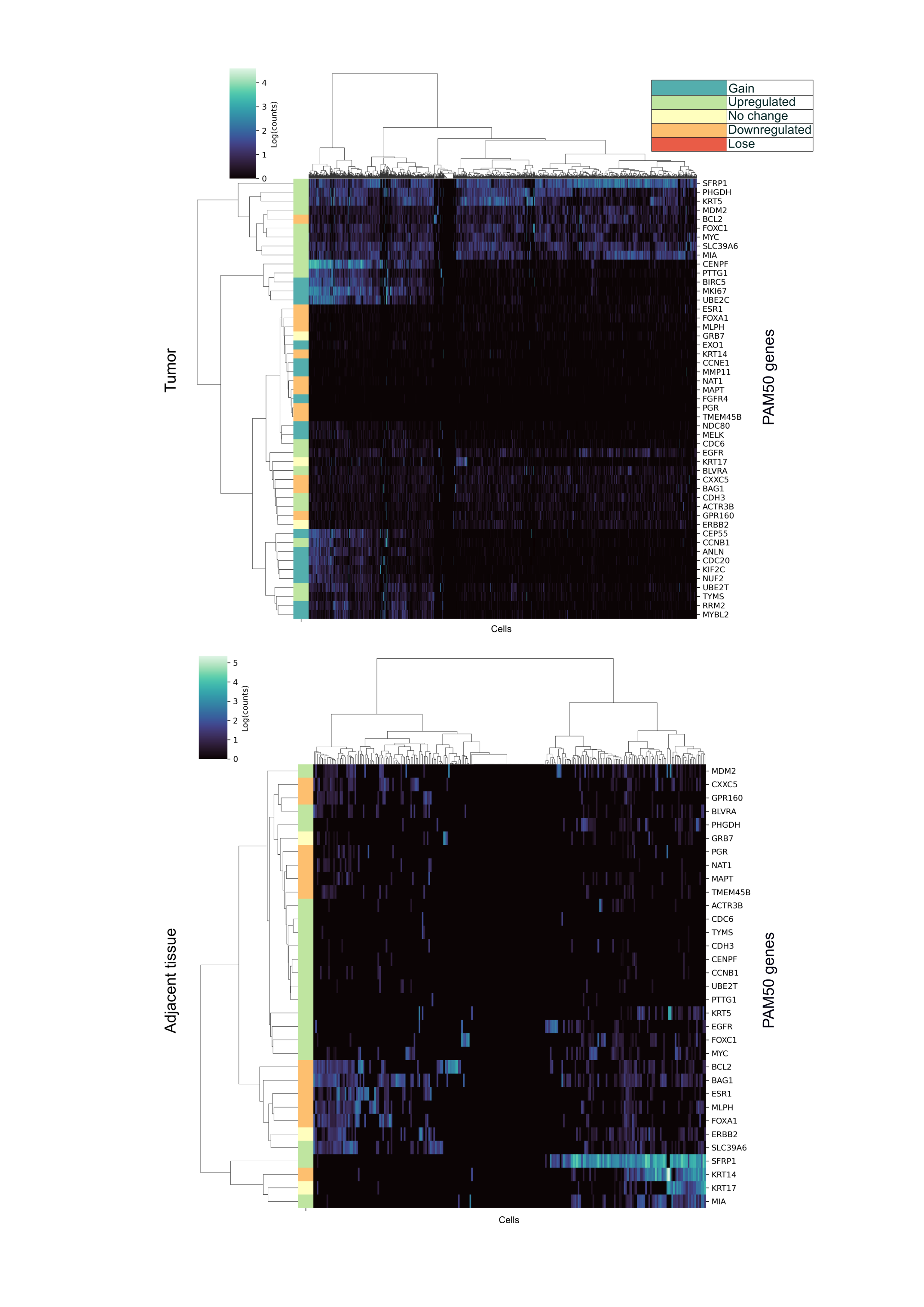

Supplement: Supplementary file 7 [file Image2.TIFF]

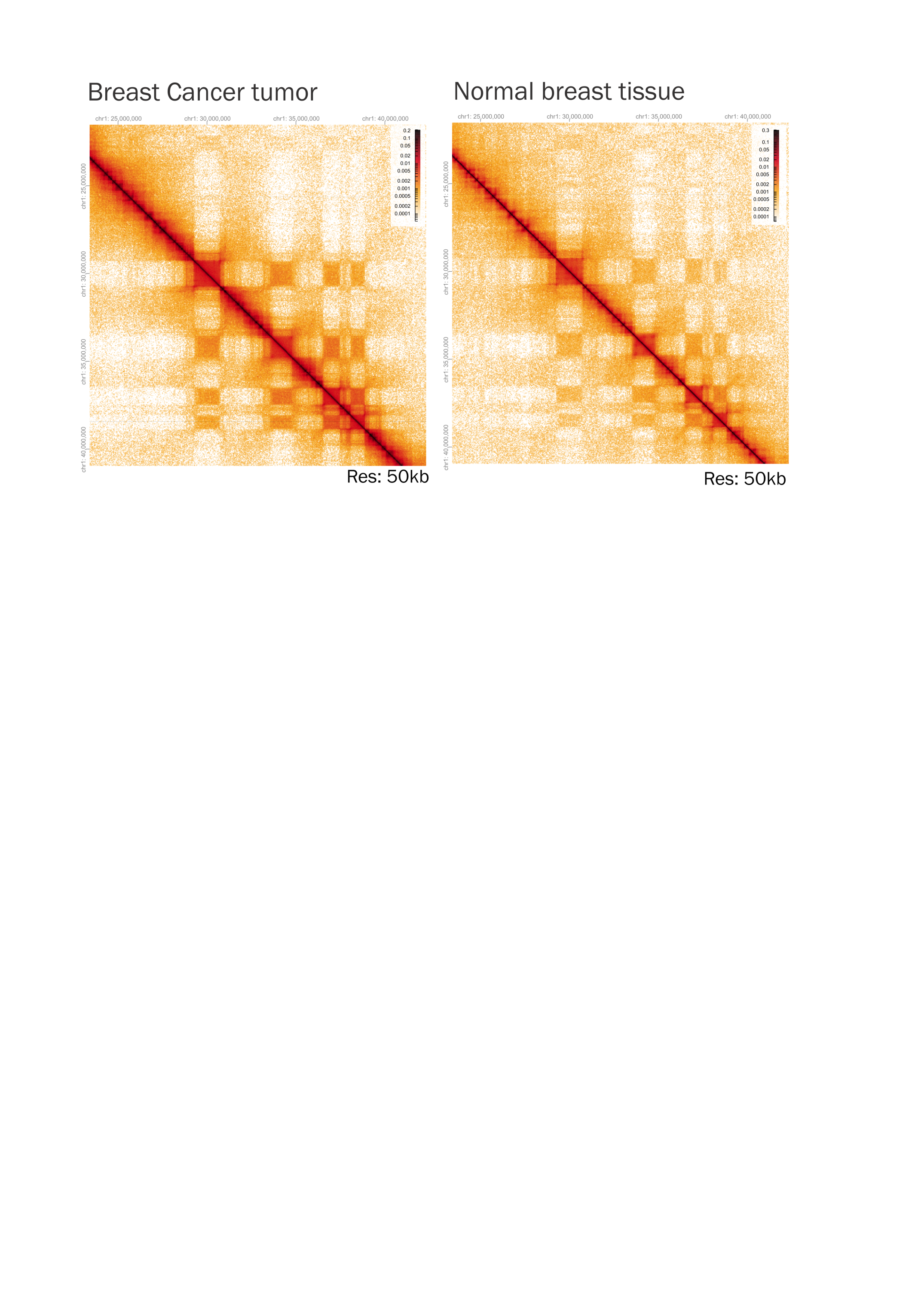

Supplement: Supplementary file 8 [file Image4.TIFF]
