# Supplementary material for: Obtention of viable cell suspensions from breast cancer tumor biopsies for 3D chromatin conformation and single-cell transcriptome analysis
Source: Front Mol Biosci. 2024 Aug 22;11:1420308. doi: 10.3389/fmolb.2024.1420308 (PMC11375512; doi:10.3389/fmolb.2024.1420308)
Supplement: Supplementary file 9 [file DataSheet1.DOCX]

**Tissue collection and transportation**

1. Collect the tissue from the primary breast cancer tumor.

- For surgery:
- Primary tissue was collected from surgical procedures in female patients without any previous treatment. The quantity of the tissue was variable depending on tumor size.
- For biopsy:
- Primar tissue was collected using TruCut needles (BIOCORE MG 12G x 10CM, Histo) taking three samples per patient consisting of approximately 1cm each.

1. Place the tissue in 5ml of RMPI 1640 medium enriched with 10% fetal bovine serum (FBS, Gibco) and 1% ampicillin/streptomycin (Biowest).
2. Transport the sample in ice to the laboratory.
3. Upon arrival weigh the sample and cut it into 1mm^3^ with surgical scissors. be careful not to crush the tissue.
4. Place the pieces in 500 µl freezing medium containing 90% FBS (Gibco) with 10% dimethyl sulfoxide (DMSO, Sigma) in a 1.8 ml cryovial (Thermo Scientific).
5. Freeze the tissue using Mr. Frosty™ Freezing Container (ThermoFisher). Put the container at -80°C for 24-72 hours and then transfer to liquid nitrogen for long-term storage

**Thaw and dissociation**

1. Thaw the tissue using a pre-heated bath at 37°C for 5-10 seconds.
2. Using a 1000 wide-bore pipette tip, carefully transfer the sample to a 15ml tube. Add 14ml of pre-warmed 10% FBS-RPMI dropwise mixing gently and continuously.
3. Centrifuge the sample at 300x g for 10 minutes. Discard the supernatant and add 1 ml of PBS to the tissue.
4. Transfer the sample to a 1.5ml tube and let it settle for 2 minutes on ice. Remove the supernatant and add 1 ml of PBS again for a second wash.
5. Carefully remove the PBS and add Accumax solution (Sigma) 100 µl per 0.01 g of tissue (Tissue must be covered by the solution). Incubate for 30 minutes at room temperature. Mix by gently tapping every 10 minutes.
6. Add 1 ml of 10% FBS-RPMI.
7. Using a 1000 wide-bore pipette tip, transfer the sample to a 40 µl filter and gently filter it with a syringe plunger making small circles (don´t crush the tissue). Wash the filter if necessary to recover all the cells.
8. Centrifuge the cells at 300x g for 100minutes at 4°C. Discard the supernatant.

**Dead cell removal and measurement of cell viability**

1. Resuspend the cell pellet in 30 µl 10% FBS-RPMI. Asses the cell viability using Trypan Blue (Sigma) and a hemocytometer.
2. Centrifuge the sample at 300x g for 10 minutes. Discard the supernatant.
3. Resuspend the cell pellet in 100 µl Dead Cell Removal MicroBeads (Dead Cell Removal Kit (Miltenyi Biotec #130090101)), and mix gently by pipetting. Incubate at room temperature for 15 minutes.
4. Add 400 µl of 1X Binding Buffer (Binding Buffer Stock Solution 20X) to achieve a total volume 0f 500 µl.
5. Place the MS Columns with magnetic beads (Miltenyi Biotec #130042201) in the magnetic field of the MiniMACS Separator (Miltenyi Biotec #130042102). Wash the column with 500 µl 1X Binding Buffer.
6. Place the cell suspension in the column and recover it in a 15ml tube. Wash the column 4 times using 500 µl 1X Binding Buffer.
7. Centrifuge the sample at 300x g for 10 minutes a 4°C. Discard supernatant.
8. Calculate cell viability by Trypan Blue. If viability is > 80% proceed with protocols, if not, process the sample again using a new MS column of the Dead Cell Removal Kit.
9. Divide cell suspension into two parts.

**Hi-C**

**Fixation and storage**

1. Take the Hi-C-destined cell suspension and bring the cell suspension to a volume of 437.5 μl with RPMI/10% FBS at room temperature.
2. Add 62.5 μl of formaldehyde (16% Agar Scientific, for a final concentration of 2%), and incubate for 10 minutes at room temperature with gentle rotation on a rocker.
3. Add 74 μl of cold 1M glycine (0.125 M final concentration). Mix by inverting the tubes.
4. Incubate for 5 minutes at room temperature followed by 15 minutes on ice.
5. Centrifuge at 400 x g for 10 minutes at 4°C.
6. Discard the supernatant, and gently resuspend the cell pellet in 500 μl of cold 1X PBS.
7. Centrifuge at 400 x g for 10 minutes at 4°C, then carefully discard the supernatant.
8. Flash freeze the pellet in liquid nitrogen and storage at -80°C.

**Lysis/Restriction Enzyme Digestion/ DNA Biotinylation**

Prepare Lysis buffer (5 ml):

4.790 ml of distilled water

50 μl of 1M Tris-HCl pH 8 (10 mM final)

50 μl of 20% NP-40/IGEPAL (0.2% final)

10 μl of 1M NaCl (10 mM final)

100 μl of complete tablet suspension 50X (protease inhibitors)

1. Resuspend the pellet in 500 μl of cold lysis buffer by gently pipetting.
2. Incubate on ice for 30 minutes, mixing by gently tapping with your fingers every 5 minutes.
3. Centrifuge the nuclei at 300 x g for 10 minutes at 4°C.
4. Resuspend the pellet in 500 μl of cold 1X NEB 3.1 buffer.
5. Centrifuge at 300 x g for 10 minutes at 4°C.
6. Prepare 300 μl of 0.3% SDS in 1X NEB 3.1 buffer (9 μl of 10% SDS in 291 μl of 1X NEB 3.1).
7. Resuspend the nuclei in 50 μl of the prepared buffer and incubate at 37°C without agitation for 45 minutes.
8. Stop the reaction by adding 25 μl of 10% Triton X-100 (final concentration of 1.14%) and 145 μl of water. Incubate at 37°C with 650 rpm for 45 minutes.
9. Add 20 μl of 10X NEB 3.1 buffer and 50U of DpnII, and incubate overnight at 37°C with gentle rotation (650 rpm).
10. Add 75U of DpnII and incubate for an additional 4 hours at 37°C with gentle rotation (650rpm).
11. Inactivate the enzyme by incubating at 62°C for 20 minutes.
12. Place the tubes on ice.
13. Add 18.75 μl of biotin-dATP (0.4 mM stock), 0.75 μl of each of the non-biotinylated nucleotides (10 mM stock) (excluding ATP), and 8 μl of Klenow LF polymerase. Incubate at 37°C with gentle rotation for 75 minutes.

**Proximity Ligation/** **Crosslink Reversion**

1. Add: 525 μl of water, 100 μl of T4 DNA ligase buffer (10X), 80 μl of Triton X-100 (10% stock), 12 μl of BSA (20 mg/ml stock), 10 μl of T4 DNA ligase (5U/μl stock). A master mix can be made without the enzyme if multiple samples are available.
2. Mix by inversion and incubate at 20°C overnight with gentle rotation.
3. Add 40 μl of proteinase K (10 mg/μl) and 2 μl of RNase A (10 mg/μl).
4. Incubate at 37°C for two hours, 55°C for one hour, and overnight at 65°C without agitation.

**DNA purification**

1. Divide the sample into two tubes, each with 500 μl.
2. Add 500 μl of Phenol/Chloroform-Isoamyl alcohol (PCI 25:24:1) and mix by vortexing for 1 minute.
3. Centrifuge the samples at 15,000 x g for 10 minutes at room temperature.
4. Perform an additional extraction by adding 100 μl of TLE and repeating steps
5. Transfer the aqueous phase (top layer) to a 2 ml low bind tube.
6. Add 55 μl (0.1X volumes) of 3M Sodium Acetate pH 5.2, 4 μl of glycogen (20 mg/ml), and 1100 μl of pure ethanol (2X volumes). Ensure the volumes are correct based on the original sample volume.
7. Mix by inversion and incubate at -20°C overnight.
8. Centrifuge the samples at 15,000 x g at 4°C for a minimum of 30 minutes.
9. Discard the supernatant and wash the pellet twice with 800 μl of cold 70% ethanol (take care not to lose the pellets). Centrifuge for 10 minutes at 4°C at maximum speed between washes.
10. Remove as much ethanol as possible with a pipette and allow the pellets to air dry for no more than 5 minutes.
11. Dissolve in 30 μl of TLE (can be incubated at 37°C for 5 minutes to promote pellet dissolution). Quantify the obtained DNA using Qubit.

**Sonication**

1. Bring all samples to a volume of 130 μl with TLE.
2. Sonicate the samples using a Covaris sonicator with the following program:

Water level: 15 ml

Duty factor: 10%

Peak Incident Power (w): 140

Cycles per Burst: 200

Time: 55 seconds

**Biotin** **Pulldown**

Prepare:

TB Buffer (10ml): 5mM Tris-HCl pH8.0, 0.5mM EDTA, 1M NaCl, 0.05% Tween)

0.5X TB Buffer (10ml)

2X NTB Buffer (1ml): 10 mM Tris-HCl pH8.0, 1mM EDTA, 2M NaCl)

1X NTB Buffer (10ml)

Note:

All washes refer to:

Add the indicated volume of buffer.

Vortex gently for a few seconds to dissolve the beads.

Rotate for 3 minutes on a rocker.

Place the sample on the magnet and remove the supernatant.

1. Wash the beads twice with 400 µl of Tween Buffer (TB). Use130 µl of Dynabeads MyOne Streptavidin C1 beads (Invitrogen) per sample.
2. Resuspend the beads in 130 µl of 2X No Tween Buffer (NTB).
3. Combine 130 µl of the library with 130 µl of beads. Incubate at room temperature for 30 minutes with gentle rotation.
4. Place the tubes on the magnet, remove the supernatant, and wash the beads twice with 400 µl of 1X NTB. (Store the supernatant as "unbound" at -20°C).
5. Wash the beads with 400 µl of 0.5X TB, incubating at 55°C for 3 minutes at 750 rpm.
6. Wash the beads with 100 µl of 1X ligation buffer (NEB).

**Biotin Removal/** **End Repair/** **A-tailing**

1. Resuspend the beads in a mixture of:

6 µl of 10X ligation buffer

2 µl of 10mM dATP

5 µl of T4 DNA polymerase

45 µl of water.

1. Incubate at 20°C for 30 min without rotation.
2. Add end repair mixture to the library:

5 µl of 10mM dNTPs

6 µl of 10X ligation buffer

5 µl of T4 PNK (10U/µl)

1 µl of Klenow LF polymerase

43 µl of water.

1. Incubate at 20°C for 30 min.
2. Wash the beads with 400 µl of 0.5X TB, incubating at 55°C for 3 min with rotation at 750rpm.
3. Wash the beads with 100 µl of 1X NEB2.
4. Add the mixture of:

5 µl of 10mM dATP

10 µl of 10X NEB2

5 µl of Klenow exo- Polymerase

80 µl of water.

1. Incubate at 37°C for 30 min without rotation.
2. Remove the supernatant and wash the beads twice with 400ul of 0.5X TB, incubating at 55°C for 3min with rotation at 750 rpm.
3. Wash the beads with 400 µl of 1X NTB.
4. Wash the beads with 100 µl of 1X ligation buffer.
5. Resuspend the beads in 50 µl of 1X ligation buffer and transfer to a new low bind tube.

**Adapter Ligation**

1. Add 4 µl of True-Seq Universal adapter with the appropriate barcode if necessary (15uM stock) and 2.4 µl of T4 DNA ligase (5U Weiss/µl). Incubate at room temperature for 2 hours.
2. Place the sample on the magnet, remove the supernatant, and wash the beads twice with 400 µl of TB 1X.
3. Wash with 200 µl of NTB 1X.
4. Wash with 100 µl of NEB2 1X.
5. Resuspend in 50 µl of NEB2 1X buffer.

**PCR Library Amplification**

1. Divide the entire library into 10 PCR reactions, each with 5 µl of beads.
2. Perform 4 cycles PCR with:

5 µl HiC beads

2 µl TS PCR primer 1 (10uM)

2 µl TS PCR primer 2 (10uM)

1.2 µl dNTPs Mix (10mM)

10 µl NEB Phusion 5X Buffer

29.2 µl water

0.6 µl NEB Phusion Polymerase

Note: The number of cycles can be set testing with 3 PCR reactions before library amplification (25 µl): 5,6,7 cycles for libraries with more than 150ng of DNA, 7,8,9 cycles for libraries with 50-150ng and 10,11,12 cycles for libraries with less than 50ng.

**Size selection**

1. Combine all the reactions into a single tube.
2. Collect the beads and the supernatant separately (SAVE BOTH). Wash the beads according to steps 58-61 of this protocol. Store in NEB2 buffer at 4°C.

For the supernatant containing the library:

1. For the selection of large fragment sizes, add 275 µl of SPRI beads (0.55X volumes).
2. Mix with the pipette 10-15 times and incubate at room temperature for 5 minutes.
3. Separate on the magnet, and allow the solution to become completely clear (approximately 5 minutes).
4. Take the supernatant and place it in a new tube.
5. Concentrate 200 µl of SPRI beads per sample to a total of 110µlvolume.
6. Add 100 µl of concentrated SPRI beads for a total of 0.75X volumes of SPRI bead buffer in each sample. Calculate according to the volume of the reactions.
7. Mix with the pipette 10-15 times and incubate at room temperature for 5 minutes.
8. Separate on the magnet.
9. Add 800 µl of 80% ethanol to wash the beads, without removing them from the magnet. Incubate for 30 seconds.
10. Remove the ethanol and repeat the wash.
11. Remove as much ethanol as possible. Let the beads dry for 2 minutes.
12. Elute with 30 µl of TLE. Incubate for 5 minutes.

**scRNA-seq**

Use the kit Chromium Next GEM Single Cell 3ʹ Reagent Kits v3.1 (Dual Index) provided by 10X Genomics. Follow the manufacturer's instructions with the next annotations on account.

1. Take the scRNA-seq-destined cell suspension (maximum 16,600 cells) and bring them to an appropriate volume according to the table provided in the manufacturer's instructions.
2. When loading Chromium NextGEM Chip G be sure to pipette the right volumes mixing well (15 times) and without introducing bubbles. If bubbles are present, eliminate them with a sterile needle.
3. Recovered libraries must be the same volume and must have a homogeneous white color. If not, it is an indication of a problem in the chip run.
